# Defining the phylogenetics and resistome of the major Clostridioides difficile ribotypes circulating in Australia

**DOI:** 10.1099/mgen.0.001232

**Published:** 2024-05-08

**Authors:** Keeley O’Grady, Stacey Hong, Papanin Putsathit, Narelle George, Christine Hemphill, Peter G. Huntington, Tony M. Korman, Despina Kotsanas, Monica Lahra, Rodney McDougall, Andrew McGlinchey, Avram Levy, Casey V. Moore, Graeme Nimmo, Louise Prendergast, Jennifer Robson, David J. Speers, Lynette Waring, Michael C. Wehrhahn, Gerhard F. Weldhagen, Richard M. Wilson, Thomas V. Riley, Daniel R. Knight

**Affiliations:** 1 Centre for Biosecurity and One Health, Harry Butler Institute, Murdoch University, Murdoch, Western Australia, Australia; 2 Communicable Disease Control Directorate, WA Department of Health, East Perth, Western Australia, Australia; 3 School of Medical and Health Sciences, Edith Cowan University, Joondalup, Western Australia, Australia; 4 Pathology Queensland, Royal Brisbane and Women’s Hospital, Herston, Queensland, Australia; 5 Melbourne Pathology, Collingwood, Victoria, Australia; 6 Department of Microbiology, NSW Health Pathology, Royal North Shore Hospital, St Leonards, New South Wales, Australia; 7 Monash University, Monash Health, Clayton, Victoria, Australia; 8 Monash Infectious Diseases, Monash Health, Monash Medical Centre, Clayton, Victoria, Australia; 9 Department of Microbiology, The Prince of Wales Hospital, Randwick, New South Wales, Australia; 10 Sullivan Nicolaides Pathology, Taringa, Queensland, Australia; 11 Department of Microbiology, PathWest Laboratory Medicine WA, Nedlands, Western Australia, Australia; 12 Microbiology and Infectious Diseases Laboratories, SA Pathology, Adelaide, South Australia, Australia; 13 School of Medicine, The University of Western Australia, Nedlands, Western Australia, Australia; 14 Douglass Hanly Moir Pathology, Macquarie Park, New South Wales, Australia; 15 Australian Clinical Labs, Microbiology Department, Wayville, South Australia, Australia; 16 School of Biomedical Sciences, The University of Western Australia, Nedlands, Western Australia, Australia

**Keywords:** *Clostridioides difficile*, WGS, microbial genomics, AMR, epidemiology

## Abstract

*Clostridioides difficile* infection (CDI) remains a significant public health threat globally. New interventions to treat CDI rely on an understanding of the evolution and epidemiology of circulating strains. Here we provide longitudinal genomic data on strain diversity, transmission dynamics and antimicrobial resistance (AMR) of *C. difficile* ribotypes (RTs) 014/020 (*n*=169), 002 (*n*=77) and 056 (*n*=36), the three most prominent *C. difficile* strains causing CDI in Australia. Genome scrutiny showed that AMR was uncommon in these lineages, with resistance-conferring alleles present in only 15/169 RT014/020 strains (8.9 %), 1/36 RT056 strains (2.78 %) and none of 77 RT002 strains. Notably, ~90 % of strains were resistant to MLS_B_ agents *in vitro*, but only ~5.9 % harboured known resistance alleles, highlighting an incongruence between AMR genotype and phenotype. Core genome analyses revealed all three RTs contained genetically heterogeneous strain populations with limited evidence of clonal transmission between CDI cases. The average number of pairwise core genome SNP (cgSNP) differences within each RT group ranged from 23.3 (RT056, ST34, *n*=36) to 115.6 (RT002, ST8, *n*=77) and 315.9 (RT014/020, STs 2, 13, 14, 49, *n*=169). Just 19 clonal groups (encompassing 40 isolates), defined as isolates differing by ≤2 cgSNPs, were identified across all three RTs (RT014/020, *n*=14; RT002, *n*=3; RT056, *n*=2). Of these clonal groups, 63 % (12/19) comprised isolates from the same Australian State and 37 % (7/19) comprised isolates from different States. The low number of plausible transmission events found for these major RTs (and previously documented populations in animal and environmental sources/reservoirs) points to widespread and persistent community sources of diverse *C. difficile* strains as opposed to ongoing nationwide healthcare outbreaks dominated by a single clone. Together, these data provide new insights into the evolution of major lineages causing CDI in Australia and highlight the urgent need for enhanced surveillance, and for public health interventions to move beyond the healthcare setting and into a One Health paradigm to effectively combat this complex pathogen.

## Data Summary

Illumina sequence data have been submitted to the European Nucleotide Archive (ENA) under study PRJEB41588 (accessions ERR5166121-5166402). Supplementary data including a detailed summary of strains and associated epidemiological data, along with sequence accessions, are hosted at Figshare (https://doi.org/10.6084/m9.figshare.20380185.v1).

**Impact Statement**

New interventions for *Clostridioides difficile* infection (CDI) rely on an understanding of the evolution and epidemiology of the circulating strains. Here we provide new insights into the genomic epidemiology of ribotypes 014/020, 002 and 056, the three most prominent *C. difficile* strains causing CDI in Australia. Utiliszing whole genome sequence data from 282 *C. difficile* isolates, we characteriszed the genetic diversity, transmission dynamics and antimicrobial resistance (AMR) repertoire of this important pathogen. Core genome analyses revealed these Australian *C. difficile* lineages are genetically diverse and widely distributed, with only limited evidence of clonal groups of strains disseminated across different States of Australia and spread over long periods. This suggests substantial and widespread sources/reservoirs of C. difficile in the community setting rather than persistent nationwide healthcare outbreaks dominated by a single clone. AMR was uncommon and incongruence between genotype and phenotype was observed for some antimicrobials. This study provides a comprehensive snapshot of the genomic epidemiology of this important One Health pathogen in Australia and highlights the need for enhanced surveillance and public health interventions to move beyond the healthcare setting and into a One Health paradigm to effectively combat this pathogen.

## Introduction

*Clostridioides* (*Clostridium*) *difficile* infection (CDI) causes life-threatening diarrhoea and is a leading cause of both healthcare- and antimicrobial-associated diarrhoeal infections in the world [1]. The US Centers for Disease Control and Prevention ranks *C. difficile* as an urgent antimicrobial resistance (AMR) threat costing the US healthcare system ~USD1 billion annually [2]. Each year in Australia, there are ~8500 cases of CDI costing $76–114 million ($13 000–19 000 per case) [3,4]. Epidemiological typing and tracking of *C. difficile* transmission within healthcare settings is key to the prevention and control of CDI. As seen with the SARS-CoV-2 pandemic, whole-genome sequencing (WGS) can identify ‘cryptic’ transmission networks undetected by conventional typing methods, but, in contrast to the UK [5], Australia has no national genomic-based CDI surveillance. Thus, our understanding of the changing epidemiology of CDI is incomplete and our ability to prevent CDI and respond to emerging hypervirulent strains is hindered, as seen in recent outbreaks caused by *C. difficile* PCR ribotypes (RTs) 244 [6] and 251 [7]. A better understanding of CDI transmission will lead to improved infection prevention and control, and patient management, and could identify potentially preventable CDI.

The *Clostridium difficile* Antimicrobial Resistance Surveillance (CDARS) study is an ongoing nationwide prospective study of *C. difficile* AMR and molecular epidemiology in Australia [8,10]. Since its inception in 2013, over 2100 *C*. *difficile* isolates have been collected across five States in Australia, and characterized by PCR RT, toxin profile and antimicrobial susceptibility [8,10]. The study reported here provides the first longitudinal data on the genomic epidemiology of CDI in Australia. We focus on toxigenic *C. difficile* RTs 014/020, 002 and 056, the three most prevalent strains of *C. difficile* causing CDI in Australia between 2013 and 2018, accounting for 29.4, 11.7 and 5.4 % of CDI cases, respectively [9].

## Methods

### Study population

The basis for this study was a collection of 282 strains of *C. difficile* comprising toxigenic RTs 014/020 (*n*=169), 002 (*n*=77) and 056 (*n*=36) sourced from humans with CDI in Australia between 2013 and 2018, as part of the CDARS study [9]. RTs 014 and 020 are genetically very similar and can be difficult to distinguish by PCR, and thus are often grouped together [8,9]. Specimens from private laboratories represented both community-associated cases (CA-CDI), as these facilities served patients from general practitioners (40–50 %) and aged-care facilities (1–3 %), as well as private (community) hospitals (50–60 %) [9]. Specimens from laboratories that were based in large tertiary-care medical centres (public hospitals) mainly represented hospital-associated cases (HA-CDI) but may have also included some CA-CDI cases [9]. Isolates originated from ten diagnostic microbiology laboratories across Western Australia (WA; *n*=48), Queensland (QLD; *n*=62), South Australia (SA; *n*=64), New South Wales (NSW; *n*=62) and Victoria (VIC; *n*=46). A summary of sequenced isolates and associated metadata is provided in Table S1 (https://doi.org/10.6084/m9.figshare.20380185.v1), available with the online version of this article.

### Whole genome sequencing

*C. difficile* strains were sequenced using an Illumina NovaSeq 6000 (Illumina) to an average read depth of 130×. Standard Nextera Flex paired-end read libraries were prepared using genomic DNA extracted from a 48 h blood agar culture of *C. difficile* using a QuickGene DNA tissue kit (Kurabo Industries). Reads were trimmed using TrimGalore v0.6.5 [11] and the quality of sequence reads was evaluated using FastQC v0.10.1 [12]. Species identification was verified using Kraken2 v2.0.9 [13]. Sequence data have been submitted to the European Nucleotide Archive under study PRJEB41588 (accessions ERR5166121-5166402, Table S1).

### Genotyping for MLST, AMR and toxins

Sequence reads were interrogated for multi-locus sequence type (MLST) using SRST2 v0.1.8 [14] following the scheme of Griffiths *et al.* [15]. Sequence reads were further investigated for AMR genotype (acquired AMR genes and chromosomal mutations) using the ARGannot v3 and ResFinder databases compiled within SRST2, and with AMRfinderPlus [16]. An in-house database of major *C. difficile* AMR transposons (Table S1) was used with SRST2 to determine the genomic context for AMR genes. Genomes were screened using SRST2 for loci conferring resistance to fluoroquinolones (*gyrA* and *gyrB* mutations) and rifamycins (*rpoB* mutations) using data hosted at the Comprehensive Antibiotic Resistance Database (https://card.mcmaster.ca/home). Multidrug-resistant (MDR) isolates were defined as possessing three or more AMR genes. * C. difficile* toxin genes (*tcdA*, *tcdB*, *cdtA/B*) were detected using a custom database of reference loci in AMRfinderPlus using default parameters.

### Microevolutionary analysis

To better understand the evolutionary dynamics and relatedness of isolates within each RT group, core-genome SNP (cgSNP) analysis was performed using the approach of Eyre *et al.* [17] and the variant calling pipeline Snippy v4.4.1 [18], as previously described [19]. Three new closed reference genomes for RT014 sequence type (ST)2 (CP076377), RT002 ST8 (CP076401) and RT056 ST34 (CP076376) were used for read mapping [20]. Gubbins v2.4.1 [21] was used to detect and remove recombination hotspots from core genome alignments. Final sets of concatenated cgSNPs in clonal frame were used to (i) calculate pairwise cgSNP differences between isolates (using snp-dists v0.6.3 [22]) and (ii) generate maximum-likelihood trees. Strains were determined to be clonally related if they fell within a threshold of 0–2 cgSNP difference, a threshold which is based on the predicted within-host evolutionary rate for *C. difficile* [17,23]. Trees were produced using RAxML v8.1.23 [24] with a generalized time-reversible (GTR) model of evolution and CAT approximation of rate heterogeneity, and were curated using iToL v6 (https://itol.embl.de/).

## Results

### Genetic diversity and evolutionary relationships of *C. difficile* RTs 014/020, 002 and 056 in Australia

**Fig. 1.**
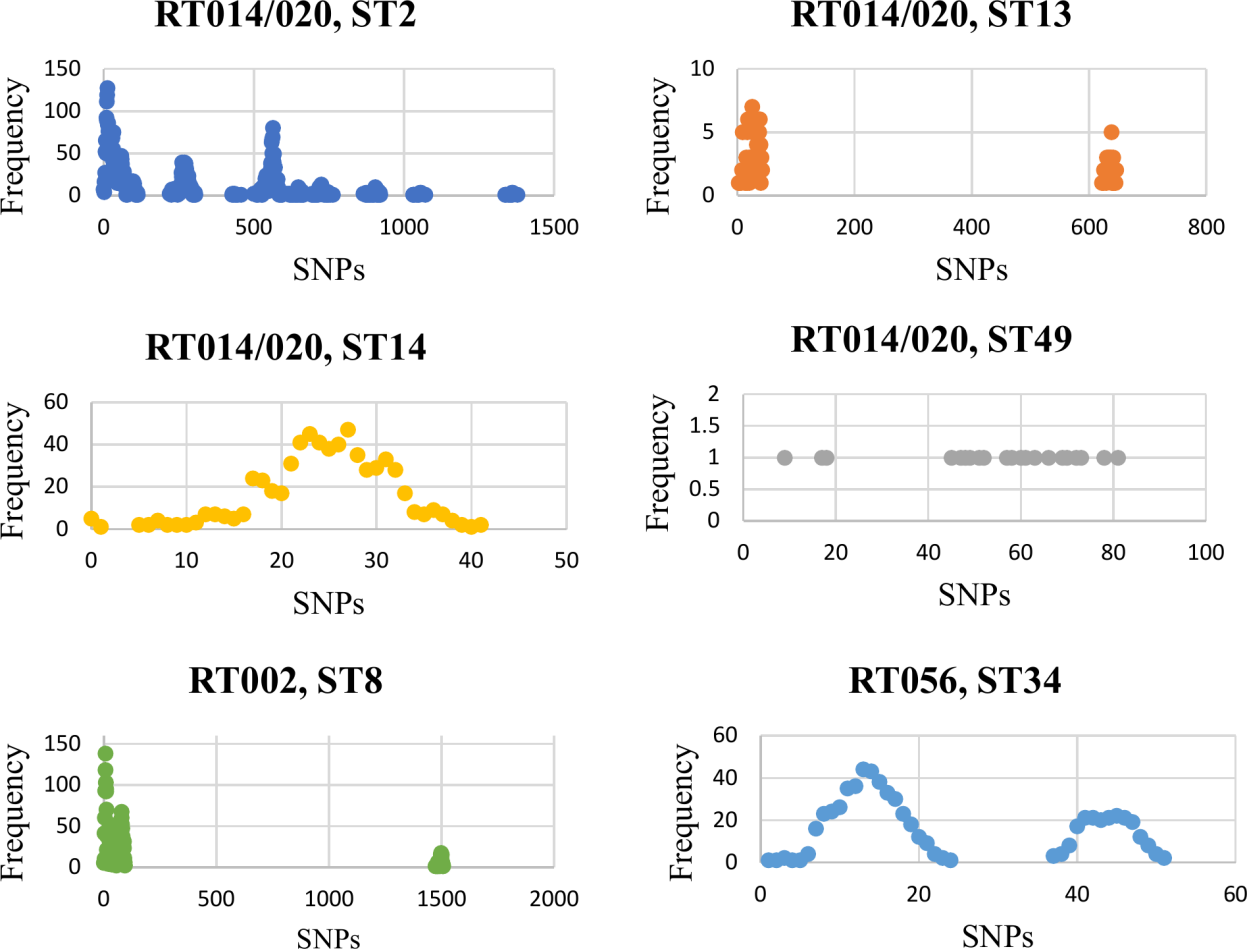
Genetic diversity of STs as measured by pairwise cgSNP distances. Each graph shows the frequency distribution of pairwise genetic distances for all strains in a given ST. STs 2, 13, 14 and 49 together comprise RT014/020, while ST8 strains belong to RT002 and ST34 strains belong to RT056.

High-resolution cgSNP analysis revealed a heterogeneous population across all three RTs, with several pairs of strains differing by >1000 cgSNPs and at most only 0.51 % of strain pairs being clonal. The average number of pairwise cgSNP differences within a RT ranged from 23.3 (RT056; ST34) to 115.6 (RT002; ST8) and 315.9 (RT014/020; STs 2, 13, 14 and 49). *C. difficile* RT014/020 was the most diverse group; almost 70 % of pairs diverged by over 100 cgSNPs and several by >1000 cgSNPs. While this was expected as RT014/020 is composed of two RTs and multiple STs, this high level of diversity was seen within ST2 also. Interestingly, different STs had different patterns of diversity as measured by cgSNP differences (Fig. 1). *C. difficile* STs 8 and 13 were characterized by two subpopulations, with strains either closely or very distantly related. *C. difficile* ST34 also had two subpopulations, but with a wider spread and more closely related strains overall. *C. difficile* ST2 featured several distinct subpopulations with large variation. *C. difficile* ST14 had a single, less distinct population while ST49 was dispersed, but both had lower variation.

**Fig. 2.**
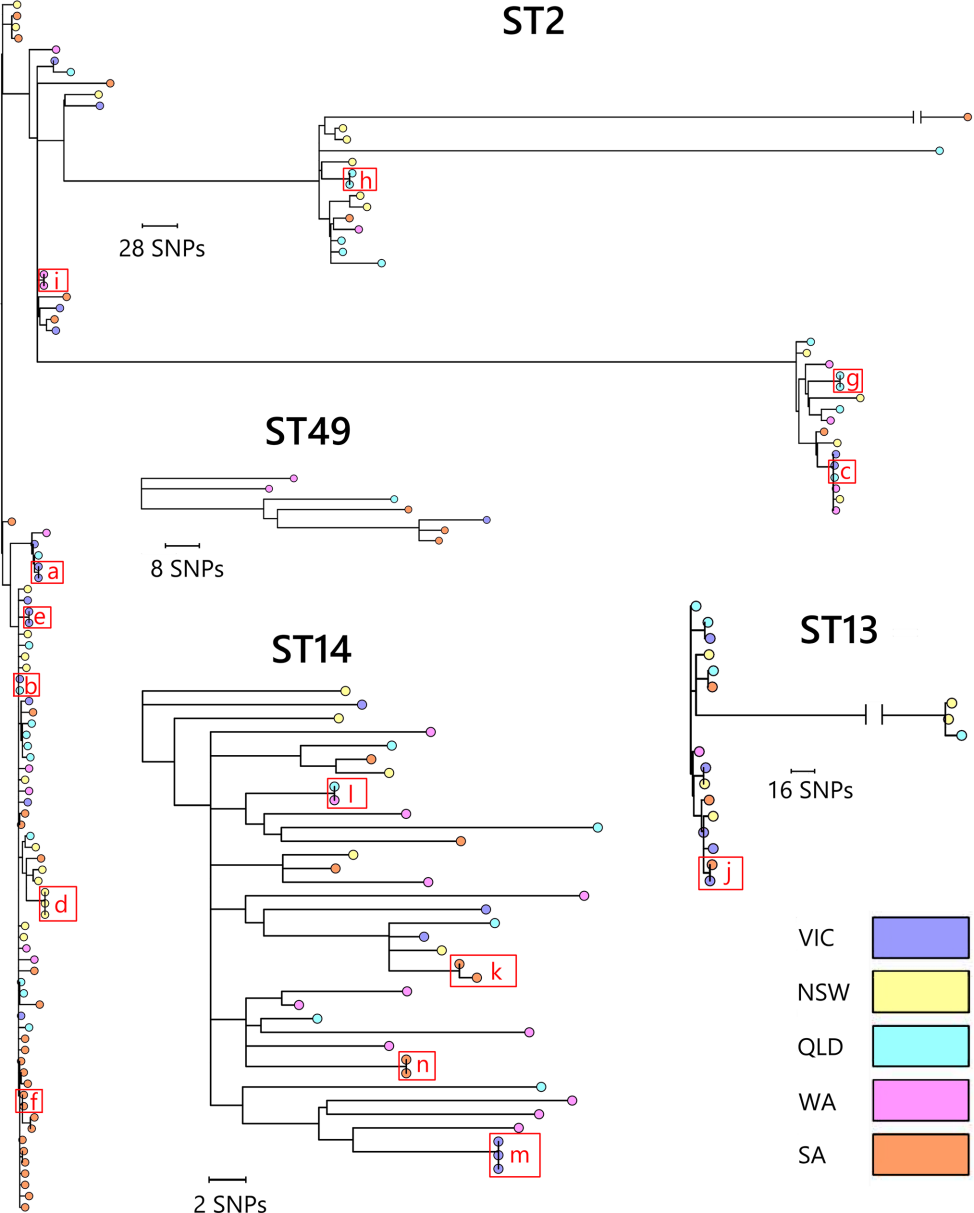
*C. difficile* RT014/020 cgSNP tree. Maximum-likelihood phylogeny of 169 *C. difficile* RT014/020 (STs 2, 13, 14, 49) genomes. Trees are based on evolution in 2716 (ST2), 1610 (ST13), 2157 (ST14) and 1275 (ST49) non-recombinant, non-repetitive cgSNPs in clonal frame. Trees are midpoint rooted, and the nodes are supported by 500 non-parametric bootstrap replicates. Tree scales are in single-nucleotide changes per quality- and recombination-filtered site. Clonal relationships (two or more strains sharing ≤2 cgSNPs) are indicated by red boxes with red letters referencing clonal strains detailed in Table 1.

**Fig. 3.**
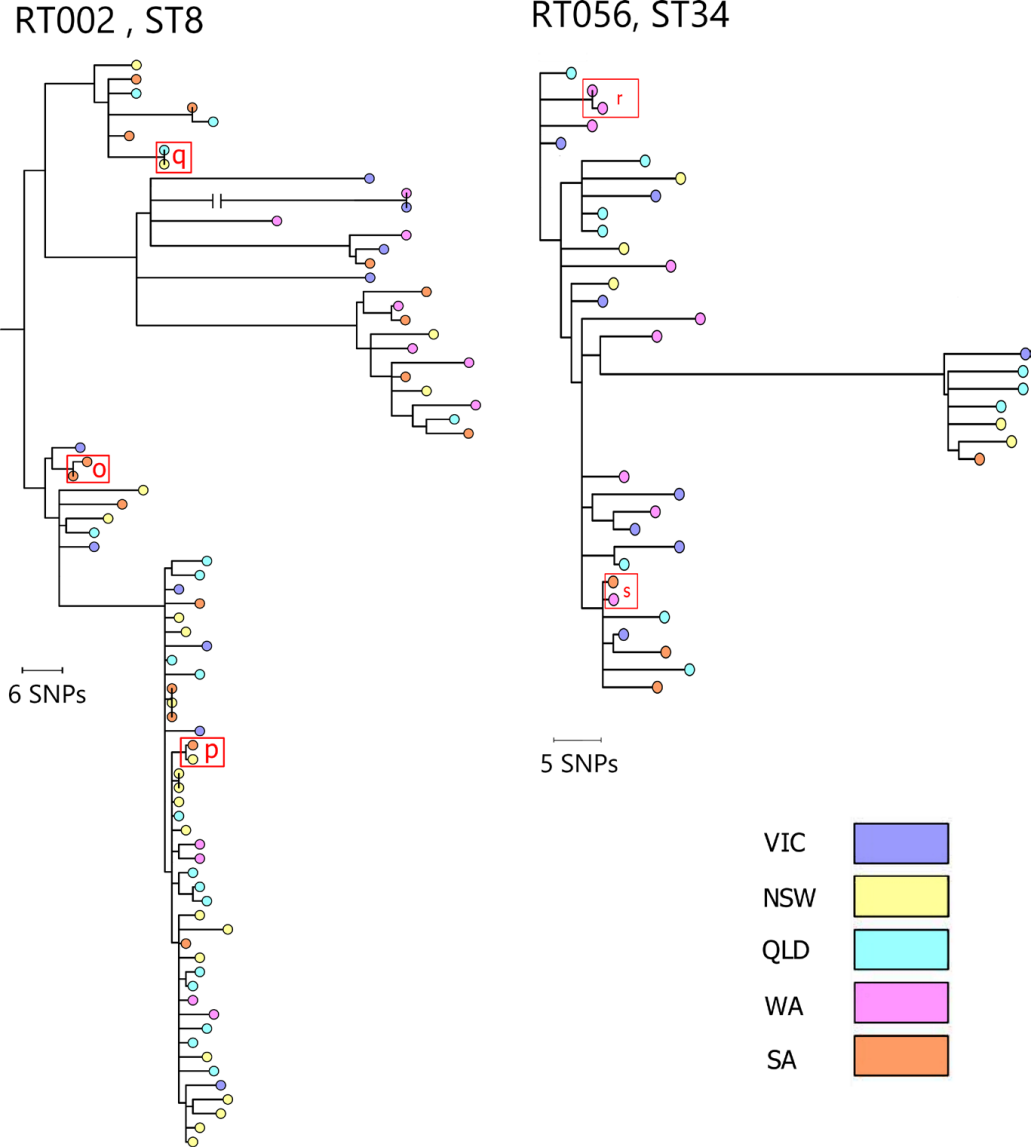
*C. difficile* RT002 and RT056 cgSNP trees. Maximum-likelihood phylogeny of 77 *C. difficile* RT002 genomes and 36 *C. difficile* RT056 genomes. The tree is based on evolution in 1955 and 9684 (RT002 and RT056, respectively) non-recombinant, non-repetitive cgSNPs in clonal frame. The tree is midpoint rooted and the nodes are supported by 500 non-parametric bootstrap replicates. Tree scales are in single-nucleotide changes per quality- and recombination-filtered site. Clonal relationships (two or more strains sharing ≤2 cgSNPs) are indicated by red boxes with red letters referencing clonal strains detailed in Table 1.

The evolutionary relationships between *C. difficile* strains within RTs 014/020, 002 and 056 are shown in Figs2  3. For RT014/020 (Fig. 2), ST14 had four clonal groups but no distinguishable large clusters, ST49 had no clonal groups or distinguishable clusters, while ST13 was split into a main group with one clonal pair and a small set of highly divergent strains. *C. difficile* ST2 was most distantly related to the remainder of RT014/020 and split into several clusters, with nine clonal groups. For RT002 (Fig. 3), approximately half the *C. difficile* strains were clustered together in a group furthest from the ancestral root. The remaining strains were found in three smaller clusters and several scattered individual strains; three clonal groups were found. Both RTs 002 and 014/020 included a small number of highly divergent isolates. *C. difficile* RT056 (Fig. 3) clustered into two groups and contained two clonal pairs. Clusters were not associated with particular Australian States; three to five States were represented in every cluster. Clusters from all RTs and STs feature strains from several different locations and collection periods, indicating that CDI in Australia is not composed of geographically distinct phylogenetic groups but rather widespread strain diversity.

**Table 1.** Summary of *C. difficile* clonal groups

Figure reference	State	Isolated at the same site	Days between isolation
**RT014/020 (ST2**)			
*a*	VIC (*n*=2)	Y	1
*b*	VIC (*n*=1) + QLD (*n*=1)	N	11
*c*	VIC (*n*=1) + QLD (*n*=1)	N	259
*d*	NSW (*n*=3)	Y	1
*e*	VIC (*n*=2)	Y	3
*f*	SA (*n*=2)	Y	168
*g*	QLD (*n*=2)	Y	0
*h*	QLD (*n*=2)	Y	39
*i*	WA (*n*=2)	N	11
**RT014/020 (ST13**)			
*j*	SA (*n*=1) + VIC (*n*=1)	N	190
**RT014/020 (ST14**)			
*k*	SA (*n*=2)	Y	1
*l*	WA (*n*=1) + QLD (*n*=1)	N	380
*m*	VIC (*n*=3)	Y	200
*n*	SA (*n*=2)	Y	3
**RT002 ST8**			
*o*	SA (*n*=2)	Y	714
*p*	SA (*n*=1) + NSW (*n*=1)	N	1432
*q*	NSW (*n*=1) + QLD (*n*=1)	N	14
**RT056 ST34**			
*r*	WA (*n*=2)	N	12
*s*	WA (*n*=1) + SA (*n*=1)	N	357

Figure references identify the letter allocated to each clonal group shown on the phylogenetic trees in Figs1^3^.

### Identification of clonal strains by cgSNP analysis and their distribution around Australia

**Fig. 4.**
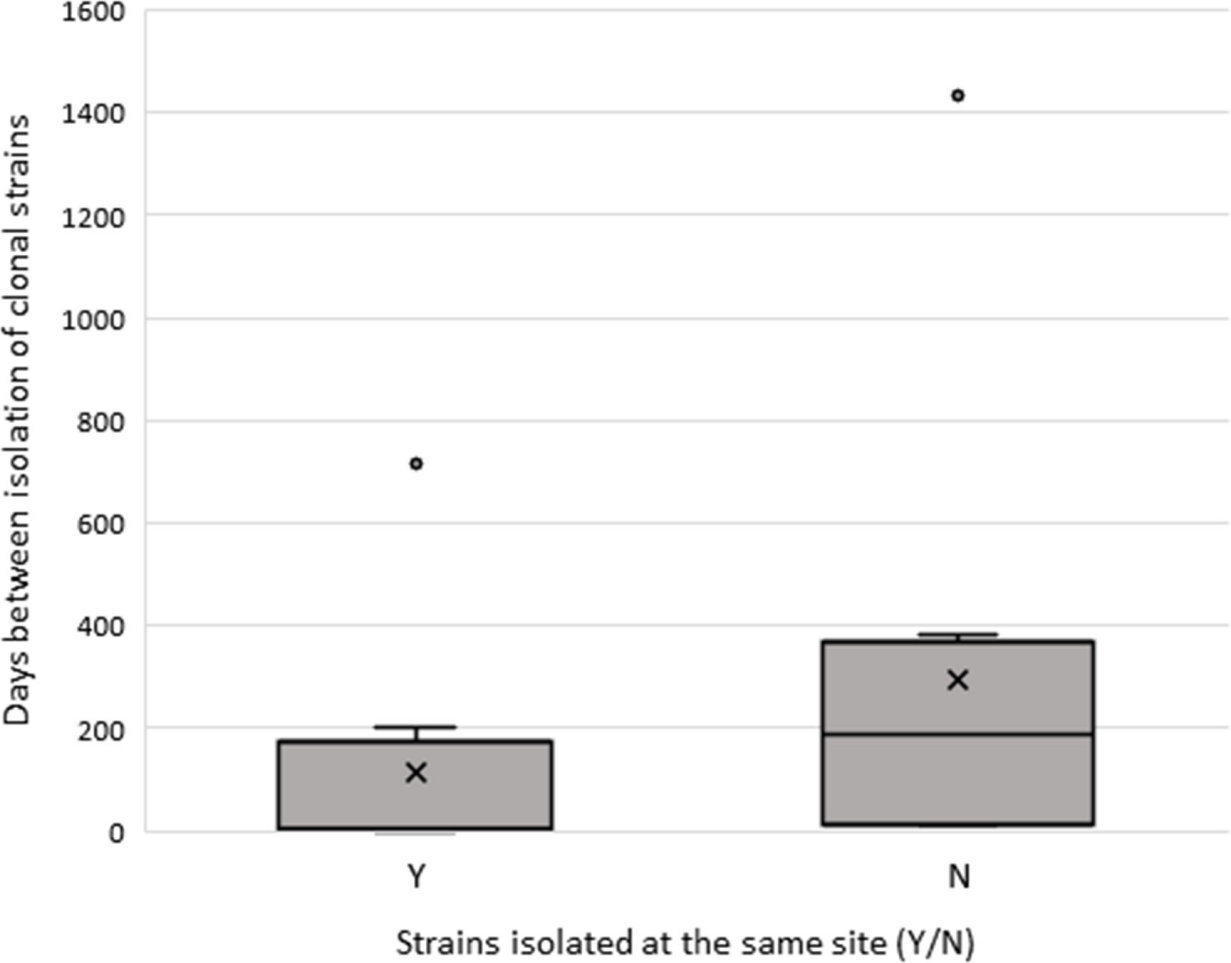
Days between the isolation of clonal pairs compared for same-site collected pairs vs. pairs collected at different sites. Outlier A consisted of two strains isolated at the same site in SA, 714 days apart, while Outlier B consisted of two strains isolated at separate sites in NSW and SA, 1432 days apart.

In total, 19 clonal groups (pairs or triplets) were detected across all RTs (RT014/020, *n=*14; RT002, *n=*3; RT056, *n=*2, Table 1). These clonal groups accounted for 17.8, 18.2 and 11.1 % of isolates within the RT014/020, RT002 and RT056 groups, respectively. Moreover, two clonal webs (defined as the occurrence of four or more strains with a clonal relationship) were identified in RT002 ST8. Clonal groups were often geographically dispersed; 63 % (12/19) were isolated within the same State, two of which were isolated at different sites, while 37 % (7/19) were isolated in different States. Same-site clonal groups were identified in all States, while cross-State clonal groups were found in VIC–QLD, VIC–SA, NSW–SA, NSW–QLD, WA–SA and WA–QLD. Both of the same-State different-site clonal groups were found in WA at hospitals within major population centres less than 200 km apart. On average, 200 days passed between the isolation of clonal strains (Fig. 4), but they tended to be found either within 2 weeks of their clones or more than 6 months apart. At the extremes, one clonal pair (RT104/020 ST2) was isolated on the same day at the same site (QLD), while another clonal pair (RT002 ST8) was isolated almost 4 years apart in different States (SA and NSW). Of the clones isolated at the same site, 60 % (6/10) were separated by less than 1 week, whereas none of those isolated at different sites were. Clones found at the same site had a mean of 113 days between the isolation of each strain, while those at different sites had a mean of 296 days. However, when outliers of 714 and 1432 days were removed, the means dropped to 46 and 154 days (same-site and different-site, respectively).

### Prevalence of AMR in *C. difficile* RTs 014/020, 002 and 056

AMR genes were detected in only 16 of 282 strains (5.7%), primarily in RT014/020 isolates, with 15 of 169 strains (8.9%) carrying at least one resistance gene. AMR prevalence varied amongst STs within RT014/020, with 5 of 108 ST2 strains (4.6%), 3 of 18 ST13 strains (16.7%) and 7 of 36 ST14 strains (19.4%) having at least one AMR gene. No resistance genes were found in RT002, while only 1 of 36 RT056 strains possessed a resistance gene (2.8%). MLS_B_ resistance was most prevalent, with 11 of 282 strains (3.9%) harbouring *ermB*, encoding a methyltransferase (Table 2). Tetracycline and aminoglycoside resistance genes were each detected in 5 of 282 strains (1.8%). For RT014/020 strains, tetracycline determinants were all *tetM* while aminoglycoside determinants included four *Aac6-Aph* alleles and one *Sat4A, Ant6-Ia, Aph3-III* combination. Interestingly, they were found only in strains with additional resistance genes, never alone. Two strains contained a non-synonymous substitution (Thr82Ile) in the quinolone-resistance-determining region (QRDR) of GyrA. No rifamycin resistance variants of *rpoB* were detected. Only a single isolate (RT014/020 ST2) was MDR, harboring *ermB*, *tetM* and *aac6-aph2* genes.

**Table 2.** AMR of Australian *C. difficile*, RTs 014/020, 002 and 056

Ribotype (*n*)	Sequence type (*n*)	MLS_B_		Tetracycline*	Aminoglycoside*	Fluoroquinolone		Rifaximin^†^
		Genotype	Phenotype	Genotype	Genotype	Genotype	Phenotype	Genotype
		*n* (%) resistant	*n* R/I/S	*n* (%) resistant	*n* (%) resistant*	*n* (%) resistant	*n* R/I/S	*n* (%) resistant
014/020 (169)	ST2 (108)	4 (3.7 %)	86/14/8	1 (4.6 %)	3 (2.8 %)	1 (0.9 %)	2/0/106	0 (0 %)
	ST13 (18)	2 (11.1 %)	16/1/1	1 (5.6 %)	2 (11.1 %)	0 (0 %)	0/0/18	0 (0 %)
	ST14 (36)	4 (11.1 %)	32/3/1	3 (8.3 %)	0 (0 %)	1 (2.8 %)	1/0/35	0 (0 %)
	ST49 (7)	0 (0 %)	6/1/0	0 (0 %)	0 (0 %)	0 (0 %)	0/0/7	0 (0 %)
002 (77)	ST8 (77)	0 (0 %)	70/4/3	0 (0 %)	0 (0 %)	0 (0 %)	2/0/75	0 (0 %)
056 (36)	ST34 (36)	1 (2.8 %)	30/4/2	0 (0 %)	0 (0 %)	0 (0 %)	0/0/36	0 (0 %)
**Total (282)**		**11(3.9 %)**	**240/27/15(85.1 %/9.6 %/5.3 %)**	**5(1.8 %)**	**5(1.8 %)**	**2(0.7 %)**	**5/0/277(1.8 %/0 %/98.2 %)**	**0(0 %)**

a*In vitro susceptibility testing was not performed for tetracycline or aminoglycosides.

b†*In vitro* rifamycin resistance was excluded from Table 2 as it showed 100 % concordance with *in silico* screening.

R, resistant; I, intermediate; S, susceptible.

### Comparison of genotypic and phenotypic AMR in *C. difficile* RTs 014/020, 002 and 056

A common phenomenon in *C. difficile*, which is especially pronounced for MLS_B_ class antimicrobials, is poor concordance between genotype and phenotype for AMR [25]. We previously conducted antimicrobial susceptibility testing *in vitro* on this set of isolates, including against clindamycin, moxifloxacin and rifamycin [10]. Only two strains had known fluoroquinolone resistance (FQR)-conferring mutations *in silico*, but testing *in vitro* detected five resistant strains (two RT002 and three RT014/020 strains) (Table 2). Both methods agreed for the two RT014/020 strains with Thr82Ile substitutions in the QRDR of GyrA. The remaining RT014/020 strain possessed an Ile139Arg substitution in GyrB that has not been identified previously as resistance-conferring. A single RT002 strain also possessed a novel substitution in GyrB, Gln434Lys, while all other RT002 strains did not contain mutations in GyrA or GyrB. No rifamycin resistance was detected either *in vitro* or *in silico*. As shown in Table 2, 140/169 RT014/020 strains were resistant to clindamycin as determined by testing *in vitro*, but only ten of these had MLS_B_ resistance determinants detected (all *ermB*). In addition, 19 strains were intermediate in susceptibility but had no resistance genes detected. While no MLS_B_ resistance genes were detected *in silico* in RT002, 70/77 were resistant and 4/77 were intermediate according to testing *in vitro*. In RT056, only a single strain had a detectable *ermB* gene (intermediate resistance), while 30/36 were resistant and 4/36 intermediate according to tests *in vitro*.

### Genetic context and architecture of AMR genes in *C. difficile* RTs 014/020, 002 and 056

AMR loci were carried on a diverse population of transposons. Nine (5.3 %) and five (2.8%) RT014/020 strains harboured transposons with *ermB* or *tetM*, respectively. These consisted of Tn*6189*, Tn*6194*, Tn*6218*, Tn*5397* and Tn*6944* (Table 3). Three strains contained two transposons and one strain that lacked transposons contained *ermB*. No transposons were detected in the RT002 group and only a single transposon (Tn*6189*) in RT056. All aminoglycoside resistance determinants were found near transposon genes. In ST2 strains, the *aac-aph* determinant was co-located with *ermB* between an IS3 family transposase IS120 and the excisionase (*xis*) and integrase (*int*) genes. Two strains in ST13 also had aminoglycoside resistance determinants, *sat4/ant6-la/aph* and *aac-aph*. In the ST13 strain with an *aac-aph* determinant, transposases from Tn*916* were found on either side of *aac-aph*, but further away and with no *int*, *xis* or *erm* genes detected. An IS66 family transposase ISSwo2 was found also close by. For the second ST13 strain, *sat4/ant6-la/aph* were found near *ermB* and an IS21 family transposase, but no *xis* or *int* genes were present.

**Table 3.** Key features and distribution of AMR transposons present in genomes of 282 *C. difficile* strains from three major RTs, 014/020, 002 and 056

**Transposon**	**Key characteristics**	**Present in**	**Reference**
**MLS**_**B**_ **resistance (** ***ermB*** * **)**			
Tn*6189*	A conjugative Tn*916-*like transposon. Transposition is due to an excisionase (*xis*) and integrase (*int*)	4 RT014/020 isolates and 1 RT056 isolate	GenBank accession MK895712.1
Tn*6194*	A conjugative Tn*916*-like transposon with a single copy of *ermB* and a putative toxin–antitoxin module. It contains *xis* and *int* genes	2 RT014/020 isolates	[48]
Tn*6218*	Related to Tn916, this transposon contains *int* and *xis* genes but is not conjugative. Some variants also contain *cfr*, *matE* and *aacA-aphD* AMR genes	3 RT014/020 isolates	[42]
**Tetracycline resistance (*tetM*)**			
Tn*5397*	A conjugative transposon closely related to Tn*916*, but transposition is due to serine recombinase (*tndX*), rather than *int* and *xis*	4 RT014/020 isolates	[49]
Tn*6944*	A recently discovered transposon commonly found in clade 2. It contains *int* and *xis* genes along with *tetM* and *mefG* (macrolide efflux) AMR genes	1 RT014/020 isolate	[40]

1*Other known *ermB*+ transposons (Tn*5398* and Tn*6190*) were not detected.

### Toxin profiles in *C. difficile* RTs 014/020, 002 and 056

All strains possessed genes encoding both TcdA and TcdB but not binary toxin, and analysis of WGS data corroborated the earlier PCR toxin gene profiling [9]. Toxin genotypes for all strains are provided in Table S1.

## Discussion

New interventions or treatments for CDI rely on an understanding of the evolution and epidemiology of the circulating strains. Our work provides new genomic insights into the epidemiology and AMR repertoire of *C. difficile* RTs 014/020, 002 and 056, the three most prominent *C. difficile* strains causing CDI in Australia between 2013 and 2018 [9]. Overall, our findings reveal that these *C. difficile* lineages are genetically diverse and widely distributed across Australia, with limited evidence of transmission between CDI cases, suggesting extensive community (and potentially animal and environmental) sources of infection rather than persistent nationwide outbreaks dominated by a single clone. AMR was uncommon and significant incongruence was observed for AMR phenotypes and genotypes.

### Australian *C. difficile* is genetically diverse and widely distributed, suggesting extensive community sources of infection

The high level of diversity and the low number of possible transmission events found in this study suggest widespread and diverse sources of infection for these major RTs, rather than persistent nationwide outbreaks dominated by a single clone. Without more detailed patient movement metadata, direct patient-to-patient transmission is difficult to prove, but the presence of clones collected at the same site within days suggests it is occurring. However, the lack of geographical clustering and the appearance of clones across States adds to the evidence that it is not the sole or a major driver of CDI as once thought. A landmark study by Eyre *et al.* used WGS to examine the epidemiology of CDI in Oxfordshire hospitals in the UK and found only ~1/3 of cases were transmissions from symptomatic patients, indicating a large proportion of cases arose from genetically diverse sources, rather than extensive transmission from a few common sources [17]. A later study by Eyre *et al.* identified two distinct patterns of *C. difficile* spread with some RTs associated with healthcare-based transmission and within-hospital clustering, while others (including RTs 014/020 and 002) were widely disseminated with less common sustained local transmission, consistent with a dominant route of transmission outside the healthcare system [26]. Our results mirror these findings of great genetic diversity and widespread sources of infection.

Environmental contamination from both human cases and animal colonization or disease could be a contributing factor. * C. difficile* has been isolated from a wide range of sources or reservoirs including wild and companion animals, food animals, plant and animal products, water sources, soils and surfaces [27,28]. Many *C. difficile* sources/reservoirs are interconnected, enabling several different transmission routes for spores. For example, fertilization of urban lawns with livestock manure may lead to the dissemination of spores into several other sources, such as local waterbodies or homes on the paws of domestic dogs [27]. *C. difficile* RT078 is common in livestock, and studies have found genetically identical strains in humans and pigs, and mechanisms of zoonotic transfer occurring with RT078 may apply to these and other strains as well [28].

*C*. *difficile* RTs 014/020 and 002 have been found in both human CDI and in animals close to people (livestock and pets) [28,29]. Our earlier work [30] found that 42 % of human strains of RT014 were clonally related to pig strains in Australia, indicating a recent evolutionary ancestry and potential interspecies transmission. Lim *et al.* [31] also suggested an origin for human CDI in pigs, as RT014/020 strains identified in compost and pigs had matching AMR patterns. Both *C. difficile* RT014/020 and RT056 have been found in/on meat products, food animals, root vegetables and lawns, all of which are fertilized with animal manure [31,32]. *C. difficile* RT002 is also common in animals in contact with humans, such as food, companion and work animals [27]. Of particular One Health concern is the potential for agricultural antimicrobial use to lead to the development of AMR in animals followed by interspecies transmission to humans. Tetracyclines, for example, are commonly used in agriculture in Australia, and both animal and human isolates of *C. difficile* RT014/020 show resistance [33].

*C. difficile* RT014/020 is one of the most commonly isolated RTs worldwide and the most common disease-causing RT in Europe and Australia [8,34]. Notably, in our previous RT014 genomic study [30], *C. difficile* clones were geographically dispersed and >50 % of cases lacked healthcare exposure. In all cases, porcine strains pre-dated human clonal strains, strongly suggesting a persistent community reservoir with long-range dissemination due to agricultural recycling of piggery effluent [30]. The recycling of effluent to agriculture and compost manufacture, leading to the dissemination of contaminated vegetables and compost in the community, demonstrates one mechanism of long-range transfer. Another was identified in a study by Thiel *et al.* who demonstrated that bacteria in manure could escape to the atmosphere during fertilization of agricultural land, and from there could be transported thousands of kilometres away [35]. In this current study, the discovery of several clonal strains in different States of Australia, thousands of kilometres apart, further adds to the evidence for long-distance transmission. All of these factors indicate that surveillance and infection prevention and control measures need to be extended beyond the healthcare system. As suggested by Eyre *et al.* [17], measures to reduce susceptibility to infection rather than reducing transmission may be more effective in Australia as well. Longitudinal genomic surveillance of CDI in the community and potential animal and environmental reservoirs needs to be implemented in Australia to better curtail this pathogen. In the past, surveillance in Australia has been fragmented and limited. While measures have been taken to improve this situation in recent years, such as the Australian Commission on Safety and Quality in Health Care (ACSQHC) [3] collecting aggregate data from healthcare facilities, there is still much to be done. A One Health approach looking into animal and environmental sources will probably be key to understanding the drivers of CDI spread in Australia.

### CDI transmission may be divided into two distinct pathways

Analysis of the geographical and temporal distance between clonal isolates demonstrated two main outcomes: clones found at the same site were generally isolated within weeks of each other, while clones found at different sites were generally isolated months apart. Our findings could be indicative of two epidemiological patterns of spread: rapid, close-range transmission with less time in spore form, or infection with dormant spores after long periods in which they may have been transmitted long distances. While it is plausible these patterns match healthcare-associated vs. CA-CDI, it is difficult to confirm without further data, especially given the issues facing classification of cases into these categories, such as underreporting of CA-CDI, varying definitions or delayed onset of symptoms masking infection sources [36,37].

### Current methods of determining clonality may be unsuitable in Australia, given the geographical and temporal dispersal of clonal strains

Eyre *et al.* [17] used SNP analysis to study >1200 isolates from symptomatic hospital patients, concluding pairs of strains differing by 0–2 SNPs and <124 days were probably a result of direct transmission, while those pairs with >10 SNPs were genetically distinct. In our study, pairwise chromosomal SNP distances of 0–2 were considered indicative of recent strain transmission events. Genetically identical strains (0 cgSNP difference) were isolated up to 4 years apart, and only 58 % (11/19) of clonal pairs were within the time frame suggested by Eyre *et al.* The molecular clock used to define evolutionary rates in *C. difficile* is based on studies of within-host strain mutation in humans. We currently do not know how quickly *C. difficile* evolves in animals or the environment, but spore formation will probably slow evolutionary rates considerably. Miles-Jay *et al.* [38] identified strain-specific differences in evolutionary rates for STs 1 (RT027) and 2 (RT014). It was speculated that this was influenced by ecological niche and selective pressures with strains adapted to healthcare settings (such as ST1) spending more time in a vegetative state, whereas strains that circulate primarily in the community (such as ST2) could spend longer in a dormant state. Therefore, the ability of *C. difficile* spores to persist for long periods in the environment in a quiescent state, and potential strain-specific differences in evolutionary rate, may mean the use of a fixed time frame for determining clonal transmission is unsuitable. More recent studies have demonstrated potential mechanisms and evidence of transmission across longer time frames. A 2019 study in a Madrid hospital used SNP analysis of strains collected over 3 years and found both long time intervals between clonal pairs (over 2 years), and a significant proportion of linked cases without direct transmission opportunities [39].

### AMR is rare and associated with mobile genetic elements in prevalent Australian RTs

AMR is a key driver of *C. difficile* epidemiology with CDI outbreaks linked to the evolution of resistance to clindamycin (RT017), fluoroquinolones (RT027) and tetracycline (RT078) [40]. Also, unlike many bacterial pathogens, acquired AMR in *C. difficile* is mediated primarily by transposons rather than plasmids [33,41]. Here we found AMR to be uncommon and largely confined to only one of the three groups examined (RT014/020) with transposons found for all except one isolate with MLS_B_ (*ermB*) or tetracycline (*tetM*) resistance. The genomic context of aminoglycoside resistance is less well defined. Dingle *et al.* [42] found that Tn*6218* variants were widespread in *C. difficile*, with some variants containing *cfr*, *matE*, and/or *aac-aph* as well as *erm* genes. In this study, the co-localization of the *ermB* and *aac-aph* determinants in aminoglycoside-resistant ST2 isolates with Tn*6218*, and the presence of nearby transposase, integrase and/or excisionase genes on the remaining aminoglycoside-resistant isolates, suggests that transposons may have played a role in the acquisition of aminoglycoside resistance, perhaps in ancestral isolates, although further research is needed.

### Genotypic and phenotypic determinations of resistance continue to be discordant

Incongruence between phenotypic and genotypic AMR is not unusual in *C. difficile*. A 2019 study of Australian *C. difficile* ST11 isolates also found that for MLS_B_ agents (e.g. clindamycin and erythromycin), AMR screening *in silico* matched poorly (36 % agreement, screened using an *ermB* gene) to agar dilution method minimum inhibitory concentration results [19]. Baines and Wilcox [43] found both clindamycin-susceptible *ermB*+ and clindamycin-resistant *ermB*− isolates in their study. MLS_B_ resistance in *C. difficile* is principally conferred by ribosomal methylation, with 23S rRNA methyltransferases most common, but other mechanisms have been implicated and much is still unknown. While the mechanism of this unorthodox resistance has not yet been determined, several possibilities have been put forward. Potential roles for efflux mechanisms and changes to 23S rDNA or ribosomal proteins were ruled out by Spigaglia *et al.* [44]. Alternative genetic determinants have been suggested and given that the majority of strains are resistant *in vitro* but susceptible *in silico* this seems likely.

The *cfr* and *cfr*-like 23S rRNA methyltransferases confer resistance to several antimicrobials in various bacterial species and have been put forward as a possible determinant [25]. However, while *cfr* has been found on *ermB*− clindamycin-resistant isolates in other studies, it was not detected here [25]. *C. difficile* RT014/020 typically has higher rates of clindamycin resistance, a risk factor for CA-CDI [31]. Clindamycin-resistant RT014/020 has been reported in animal and environmental sources such as pigs, lawns and food products [31,45]. Given the diversity and limited epidemiological links found in this study, in combination with its association with such community reservoirs, it seems likely that a significant proportion of RT014/020 cases are acquired outside the hospital system and may ultimately be traced back to animal sources.

FQR in *C. difficile* typically occurs via mutations that reduce binding affinity in the target site, the QRDR of DNA gyrase subunits A and B [25]. The Thr82Ile substitution in GyrA found in two isolates in this study is the most common resistance-conferring mutation and may be maintained in a population due to the absence of a detectable fitness cost [25]. The presence of FQR in isolates lacking *gyrA* or *gyrB* mutations is highly unusual but has been identified before in Taiwan and Ireland [46,47]. Whether any of the novel *gyrA*/*gyrB* mutations in resistant strains cause resistance, or whether there are still unknown resistance determinants in play, will require further research. Given the important role that FQR played in the spread of the well-known hypervirulent strain RT027 across North America and Europe, the elucidation of resistance mechanisms may be important to prevent future outbreaks [25]. However, FQR strains are unlikely to have the significant fitness advantage in Australia that they enjoyed in other countries given the restricted use of fluoroquinolones here [25].

We acknowledge that limited epidemiological metadata were available for analysis. Combining longitudinal tracking of CDI cases and collecting data on patient treatment, clinical outcomes, comorbidities and movement within the hospital setting and the community would allow for more conclusive identification of transmission events and designation of cases as hospital- or community-associated. Despite this, the epidemiological patterns we observed further underscore the value of integrating high-resolution genomic surveillance data with even limited epidemiological metadata.

In conclusion, genomic analysis of *C. difficile* RTs 014/020, 002 and 056 revealed a genetically diverse population with limited evidence of direct transmission and low rates of AMR. While *in silico* screening offers many benefits, the incongruence between genotype and phenotype found in this study demonstrates the need for ongoing research to identify novel determinants of resistance, as well as continued phenotypic evaluation of AMR. Epidemiological analysis of *C. difficile* is complex due to its spore-forming nature and numerous reservoirs/sources. The dispersal of clonal strains across large distances and long time frames highlights this complexity and suggests widespread sources of infection outside the healthcare system. It is becoming clear that research, surveillance, and infection prevention and control will all need to move beyond hospitals and to a One Health paradigm to effectively combat this pathogen.

## Additional information

Supplementary Data is available at https://doi.org/10.6084/m9.figshare.20380185.v1

**Impact Statement**

**New interventions for *Clostridioides difficile* infection (CDI) rely on an understanding of the evolution and epidemiology of the circulating strains. Here we provide new insights into the genomic epidemiology of ribotypes 014, 002 and 056, the three most prominent *C. difficile* strains causing CDI in Australia. Utilizing whole genome sequence data from 282 *C*. *difficile* isolates, we characterized the genetic diversity, transmission dynamics and antimicrobial resistance (AMR) repertoire of this important pathogen. Core genome analyses revealed these Australian *C. difficile* lineages are genetically diverse and widely distributed, with only limited evidence of clonal groups of strains disseminated across different States of Australia and spread over long periods. This suggests substantial and widespread sources/reservoirs of *C. difficile* in the community setting rather than persistent nationwide healthcare outbreaks dominated by a single clone. AMR was uncommon and incongruence between genotype and phenotype was observed for some antimicrobials. This study provides a comprehensive snapshot of the genomic epidemiology of this important One Health pathogen in Australia and highlights the need for enhanced surveillance and public health interventions to move beyond the healthcare setting and into a One Health paradigm to effectively combat this pathogen.**

**Impact Statement**

New interventions for Clostridioides difficile infection (CDI) rely on an understanding of the evolution and epidemiology of the circulating strains. Here we provide new insights into the genomic epidemiology of ribotypes 014, 002 and 056, the three most prominent C. difficile strains causing CDI in Australia. Utiliszing whole genome sequence data from 282 C. difficile isolates, we characteriszed the genetic diversity, transmission dynamics and antimicrobial resistance (AMR) repertoire of this important pathogen. Core genome analyses revealed these Australian C. difficile lineages are genetically diverse and widely distributed, with only limited evidence of clonal groups of strains disseminated across different States of Australia and spread over long periods. This suggests substantial and widespread sources/reservoirs of C. difficile in the community setting rather than persistent nationwide healthcare outbreaks dominated by a single clone. AMR was uncommon and incongruence between genotype and phenotype was observed for some antimicrobials. This study provides a comprehensive snapshot of the genomic epidemiology of this important One Health pathogen in Australia and highlights the need for enhanced surveillance and public health interventions to move beyond the healthcare setting and into a One Health paradigm to effectively combat this pathogen.

## Abbreviations

AMR: antimicrobial resistance
CA: community-acquired
CDI: *Clostridioides difficile* infection
cgSNP: core genome SNP
FQR: fluoroquinolone resistance
HA: hospital-acquired
MDR: multi-drug-resistant
QRDR: quinolone-resistance-determining region
RT: ribotype
ST: sequence type
WGS: whole genome sequencing
